# The theorisation of ‘best interests’ in bioethical accounts of decision-making

**DOI:** 10.1186/s12910-021-00636-0

**Published:** 2021-06-01

**Authors:** Giles Birchley

**Affiliations:** Centre for Ethics in Medicine, Population Health Sciences, Medical School, University of Bristol, Canynge Hall, 39 Whatley Road, Bristol, BS8 2PS UK

**Keywords:** Best interests, Medical law, Political philosophy, Liberalism, Shared decision-making

## Abstract

**Background:**

Best interests is a ubiquitous principle in medical policy and practice, informing the treatment of both children and adults. Yet theory underlying the concept of best interests is unclear and rarely articulated. This paper examines bioethical literature for theoretical accounts of best interests to gain a better sense of the meanings and underlying philosophy that structure understandings.

**Methods:**

A scoping review of was undertaken. Following a literature search, 57 sources were selected and analysed using the thematic method.

**Results:**

Three themes emerged. The first placed best interests within the structure of wider theory, noting relationships with consequentialism, deontology, prudential value theory, rights and political philosophy. The second mapped a typology of processes of decision-making, among which best interests was ambiguously positioned. It further indicated factors that informed best interests decision-making, primarily preferences, dignity and quality of life. The final theme considered best interests from a relational perspective.

**Conclusions:**

Characterisation of best interests as strictly paternalist and consequentialist is questionable: while accounts often suggested a consequentialist basis for best interests, arguments appeared philosophically weak. Deontological accounts, found in law and Kantianism, and theories of political liberalism influenced accounts of best interests, with accounts often associating best interests with negative patient preferences (i.e. individual refusals). There was much more emphasis on negative interests than positive interests. Besides preference, factors like dignity and quality of life were held to inform best interests decisions, but generally were weakly defined. To the extent that preferences were unable to inform decision making, decisions were either made by proxy authority or by an intersubjective process of diffuse authority. Differing approaches reflect bifurcations in liberal philosophy between new liberalism and neo-liberalism. Although neither account of authority appears dominant, bias to negative interests suggests that bioethical debate tends to reflect the widespread ascendancy of neo-liberalism. This attitude was underscored by the way relational accounts converged on private familial authority. The visible connections to theory suggest that best interests is underpinned by socio-political trends that may set up frictions with practice. How practice negotiates these frictions remains a key question.

**Supplementary Information:**

The online version contains supplementary material available at 10.1186/s12910-021-00636-0.

## Introduction

The ‘best interests’ principle is a key component of policies concerned with decision-making in every area of medical practice. The principle of informed consent suggests that persons of sound mind may refuse medical treatment because autonomous persons should decide what is best for themselves. However when autonomous persons demand treatment, their demands are ostensibly limited by what is in their best interests. Often when a patient lacks autonomy—from the barriers to communication arising from developmental immaturity in neonatal intensive care, to temporary, anaesthesia-induced, insensibility in operating theatre, to fluctuating capacity due to dementia, to irreversible loss of consciousness at the end of life—a fundamental principle governing decision-making is that a decision be made in a patient’s best interests. While, historically, ‘best interests’ can be found in rhetorical use in moral discourse going back to the eighteenth century [1–3], even rhetorical claims about best interests will be underpinned by particular theoretical assumptions. Yet the theoretical basis of best interests is unclear, and thus the role of best interests in future policy remains uncertain. This paper presents analysis suggesting that the approach taken to best interests in the bioethics literature is often reductive, focusing predominantly on negative interests in a way that suggests concordance with ideology and a potential for frictions in practice. There is more to say about best interests than much of the bioethics literature suggests.

From the perspective of English medical law, best interests is an important concept that determines medical treatment in common law [4, 5] and statute [6, 7]. However, it has international resonance too, underpinning the status of children in the UN Convention on the Rights of the Child 1989 (UNCRC) and the wellbeing of adults in international healthcare declarations [8], especially those adults not competent to make healthcare decisions [9]. Yet the role of best interest in medical law is relatively recent, entering largely due to links with parens patriae common law traditions that established custody over minors and the mentally infirm [10, 11]. Despite the relatively recent importance of best interests to medicine, the link to custody is more indicative of the wider history and context. As a legal concept of child custody, best interests is the concept by which the state exercises responsibility for the protection of children, but has thereby also been associated with the removal of children from parents deemed unfit due to poverty or sexual orientation [12, 13]. Indeed, feminist scholars argue that the concept of best interests entered statute as means of limiting the increasing common law recognition of rights of divorced mothers to have custody of their children [14]. A quite different scope is seen in the role of best interests as the underlying principle in the UNCRC, and its subsequent feature in provisions of national constitutions [15–17] and laws [7, 18–20] addressing the wellbeing of children. The advent of the UNCRC connects the concept of best interests to rights and interests of all types, including preferences, cultural autonomy, protected relationships with parents and positive rights upon the state. Best interests continues to affect a range of non-medical children’s issues in common law countries, for example relationships within religious communities and parents’ educational choices [21], and immigration rights [22] to name but a few. Thus we can see that, outside medicine, the concept of best interests (1) affects a range of actors, interposing between citizens in dispute; and between citizens and the state, using (2) approaches asserting both positive and negative rights and/or interests (which for convenience I will hereafter generally conflate as ‘interests’), for (3) a range of political or social ends including the protection or the marginalisation of unfavoured groups.

The ensuing complexity means that ‘best interests’ is both defended and vilified in bioethics. Critics argue best interests undermines patients’ liberties [23, 24] and that ‘best interests’ is so vague that it allows any arbitrary set of factors to be determinate [25–27]. This critique has particularly gained ground in two areas: disability rights, and the liberties of parents and families. The United Nations Committee on the Rights of Persons with Disabilities [28] argues that the best interests principle is incompatible with Article 12 of the United Nations Convention on the Rights of Persons with Disabilities (UNCRPD). Article 12 asserts the superiority of a ‘rights, will and preferences’ standard in upholding the right of disabled persons to have equal recognition before the law. Others [29, 30] argue that parental liberties are trampled because best interests interferes with child raising and fails to account for parental interests. These critics have often proposed an alternative standard of ‘significant harm’, which they argue better balances parental rights against the state power in children’s healthcare decisions [31, 32]. Defenders of the concept of best interests argue that it fixes attention on the most vulnerable party within a decision, and allows decision-makers to respond to individual situations with discretion [33–35]. Whatever its inadequacies, many argue that the best interests standard plays a necessary role [36, 37]. Others suggest alternative standards like ‘significant harm’ or ‘rights will and preferences’ are inadequate replacements [38, 39]. However, it is not clear in every case what either critics or defenders mean by ‘best interests’. What, if any, are the disparate and the common threads that underlie their theorisations? and how do these theorisations reflect on the different actors, approaches and ends seen more broadly in best interests in other areas? To address these questions I undertook an analysis of the legal and bioethics literature.

## Methods

The breadth of the inquiry and limitations in the time available to conduct the analysis meant that I sought to assess the shape of the literature rather than engage with every potential source. I therefore conducted a scoping review [40]. A detailed description of the search strategy and results is given in Additional file 1: Detailed Methods. Briefly, Pubmed and JSTOR databases were searched using fixed keywords that aimed to identify theoretical accounts of best interests. 659 papers were screened and 53 were identified. A further four papers containing important theoretical accounts that had not been picked up in the review were added. All papers were analysed using thematic analysis [41] with papers coded for recurring ideas and concepts using NVivo software, and these codes developed into themes.

## Results

From thematic analysis of 57 sources containing theoretical accounts, three themes emerged. The first highlights connections with theory that shed light on the concept of best interests. The second considers the processes of decision-making for others, detailing a contiuuum that spans preferences and shared decision-making approaches, encompasses several distinct approaches, and highlights factors that are argued to inform best interests decision-making. Finally, the third theme considers competition between patient-centred and relational approaches to best interests decisions. Overall the themes reveal that accounts that emphasise personal preference (or, in the last theme, proxy preference) dominate. Approaches to inaccessible preferences rely on either diffusing authority and empowering families.

### Theme 1: Best interests and theory

The first theme traces connections between best interests and recurring theoretical concepts or ideas. Theoretical discussion was not always explicit, and in some cases where theory *was* made explicit, there was little or no critical exposition. In this sense, best interests appears under-theorised. Nevertheless, accounts of best interests and adjacent concepts of consequentialism, prudential value, deontology, rights and political philosophy indicated important elements and demarcations of best interests in theory.

#### Consequentialism

Consequentialist ethical theories, including utilitarianism, are commonly associated with best interests. Consequentialist theories judge the rightness or wrongness of actions according to their consequences—in the case of consequentialism the degree an action promotes or reduces a predefined concept of good. Association with these theories can be overt, or through identifying best interests with an element of these theories, such as the aim of ‘maximising goods’.

Best interests is argued to be consequentialist because effective best interests decision-making depends on accurately predicting consequences [42] or because best interests focus on long-term goods [43]. In some cases, consequentialism is overtly identified with utilitarianism. Shewchuk [44] identifies best interests as utilitarian because of the connection of beneficence, a principle which promotes the good, balances harms and benefits and owes a large debt to utilitarianism [45]. More commonly, commentators suggest (explicitly or implicitly) that best interests involves similar considerations to utilitarianism, such as calculating welfare [46] by balancing benefits and burdens [44, 47–62] and/or maximisation of the good [46, 50, 51, 58, 60, 63–68]. The convergence on ‘maximisation’ masks some disagreement over whether maximisation implies ‘strict’ pursuit of a single good [47, 56, 59, 67, 69, 70] or a more pragmatic, all-things-considered, interpretation of good [42, 58].

#### Prudential value

Best interests are also associated with theories of wellbeing [52, 57, 71–73], often called ‘prudential value’ theories, which identify what it is that makes things go best for individuals. While these theories potentially have links to the concept of utility (identifying prudential value may on some accounts identify the overall good to be maximised in a population), they are distinct from utilitarianism.

DeGrazia [71] makes an explicit connection between best interests and three theories of prudential value: mental states (such as happiness), desires (satisfying wants) or objective lists (satisfying needs). Some consider objective lists defensible for patients whose values are unknown, like intellectually disabled children [57]. Despite the similarity of desire theories to the English legal account in the Mental Capacity Act 2005 [72], others consider all three theories advanced by DeGrazia deficient for healthcare decision-making [72, 73]. These authors disagree on whether a mixed approach should be taken drawing on all three theories on a case by case basis [72] or if a new theory is needed. Of the latter, Hall [73] proposes ‘Pragmatic subjectivism’, a theory that bases wellbeing on whichever of the patient’s own values are most stable and functional.

#### Deontology

Best interests is a legal principle, discussions of statutory and case law are frequent, and this leads to some reflection of the nature of legal reasoning (jurisprudence). Additionally, reference is made to elements of Kantianism—the moral philosophy of Immanuel Kant which describes a system by which individuals discover their duties and obligations. Thus two rule-based, or deontological, philosophies (Kantianism and jurisprudence) appear regularly in the literature.

Some sources discuss best interests and law in the language of legal duty [54, 55, 70, 74, 75], particularly identifying underlying fiduciary legal duties (duties based on status and trust) of parents and clinicians [44, 69, 74]. A range of other duties, of varying specificity, are identified, including medical duties to prolong life, promote health or relieve suffering [44, 61], respecting advance decisions [63], beneficence [76] and nonmaleficence [34], and duties to promote children’s best interests [51, 58, 65]. The frequent discussion of rights in proximity to best interests (see below) often draws Kantianism into the law by invoking dignity and autonomy [77].

Further papers refer to the Kantian injunction not merely to use persons as a ‘means to an end’ in a best interests decision. This might be applied to specific cases of benefit to others such as forbidding treating a patient solely to benefit a relative [74] or involvement of children in research and organ transplantation that will only benefit others [66, 67]. In other cases Kant’s means-end prohibition is presented in a way that may inform judgements of best interests [44]. In the context of best interests, the Kantian concept of ‘dignity’ can also inform discussion of rights [77]. As we shall see below, rights have a mixed relationship with best interests.

#### Rights

While generic concepts of human rights (or patient rights) were rarely discussed, many sources contain appeals to the rights of parents, children and persons with disabilities (the latter sometimes linked to the respective UN conventions). As a concept, ‘rights’ has a variable relationship to’best interests’. Discussions of parental rights and the rights of persons with disabilities tend to be antagonistic to the concept of best interests, while a more positive association exists between children’s rights and children’s best interests.

Considering the rights set down in the United Nations Convention on the Rights of the Child (UNCRC), Daniels et al. [78] suggest a theoretical framework that connects rights to interests. Therein a right is viewed as something that can be claimed against another to satisfy an interest, an interest is an issue of importance to an individual based on a need, and a need is an interest that is material in the world.While this sketch is rather underdeveloped,^1^ it implies the interconnection of rights and interests. For rights and interests to be connected in this way, it has been observed that we must conceive of rights based on objective interests, rather than on preferences or will [37]. Opposingly, those who argue rights have a priority over interests [77, 81] are likely to implicitly favour a ‘will’ theory of rights.^2^ While such views do not necessarily deny that there are objective goods that might constitute interests where an account of the will is absent, rights based on interests need detailed specification if they are to guide practice. Some authors argue that the relatively sparse level of detail in rights declarations makes rights undeterminative of specific legal questions [53] or of children’s clinical decisions [78, 83]. Although elaboration of rights does not appear to be an insuperable task, few overt elaborations were attempted in the literature reviewed.

Despite such scepticism of children’s rights [83] a number of commentators focus on parental rights. Some [13, 44, 84] consider parental rights in relation to custody and an unencumbered relationship with their child, while others focused on medical decision-making [44, 56, 59, 85]. Often the latter identify parental rights as rights of authoritative decision-making on behalf of their child, to which best interests are believed antagonistic [44, 56, 59]. Parental rights are said to give parental discretion a prerogative (a view examined further in theme 2) which best interests denies. Thresholds of parental discretion—below which parental liberty is curtailed—are cited frequently. While best interests itself may be taken as a threshold below which parental (or other’s) choices are scrutinised [56–58], dissatisfaction with the opacity or fairness of best interests means that harm [46, 65, 67, 77, 84, 86] and reasonableness [58, 65, 86] are more frequently cited alternatives. Distress [67], informedness [58] and basic interests [34] are also mentioned. Arguments for thresholds other than competence or capacity are (almost [63]) exclusively associated with parental decisions.^3^ Contrasting children’s and parental rights, there is a notable distinction between the positive interests to guide action that are sought (or critiqued) in accounts of children’s rights and the negative interests in liberty that are asserted in parental rights discourse.

Although several authors discuss critique of best interests from the disability rights movement there is no similar contrast therein between negative and positive interests. The critique asserts that the opacity of best interests decisions makes people with disabilities vulnerable to decisions that ignore their interests. Instead, factors such as resource limitations, third party interests [69, 77, 87] and decision-maker prejudices [42, 75] may guide best interests decisions. Defending best interests, Snelling [77] suggests that these hazards may be mitigated by foreknowledge. She and others [88] argue that best interests, by focusing on what would make a patient’s life go best, is complementary to the focus of rights on the subjective experiences of the patient.

#### Political philosophy

While several papers discuss theories of rights, other theories from political philosophy also underpin understandings of best interests, including concepts of liberty, representation, liberalism and libertarianism. Most of these accounts fall within the scope of political liberalism (if libertarianism is a form of liberalism that places greater weight on liberty rights than welfare rights).

While several authors identify best interests with paternalism [37, 75, 76, 89] and either identify anti-paternalism with Millian liberalism, Isaiah Berlin’s concept of ‘negative liberty [75] or libertarianism [76], their approaches differ. Dworkin considers the question is whether a best interests decision is envisaged to represent the wishes or the needs of the patient [89]. He argues that the answer will depend upon whether best interests are perceived as questions of technical expertise (per Burkean conservatism) or of subjective perception (per Millian liberalism). On the other hand, Archard notes that Mill’s version of absolute anti-paternalism both prohibits even very small interferences with extremely harmful decisions of adults, and is radically inconsistent with the way children’s decisions are interfered with. He therefore argues that the gap between the adult autonomy and child best interests approaches should be closed [37], ostensibly by adopting a more paternalist approach for adults and a less paternalist approach for children. Taylor [75] notes that those who lack competence (and thus, by inference, may have decisions made in their best interests) are generally denied ‘negative liberty’ (a private sphere of activity free from outside interference). Thus she argues best interests is an illiberal obstacle to the extension of the important liberties to this population. Welie [76], identifying best interests with beneficence, suggests that the common precedence given to anti-paternalist autonomy over beneficence is libertarian in origin. The emphasis on non-interference in medical decisions means that participants must take personal responsibility for whatever outcome occurs, allowing the state to wash its hands of matters deemed beyond its control, such as the impact of patient’s differing natural abilities to meet their own needs.

Finally, Lim et al. [87] consider the process by which best interests are decided, by focusing on how best interests can accommodate value pluralism in a liberal state. Explicitly rejecting Rawls’ [90] theory that there is an ‘overlapping consensus’ where different comprehensive doctrines meet, the authors reject the contention that a comprehensive set of principles for best interests can be drawn. Arguing that no common ground about what counts as a ‘good life’ exists in modern liberal society, the authors maintain that only an ‘enumerative’ approach, where individual decision-makers follow discretionary and underspecified principles, is compatible with a liberal state. Such radical value pluralism severely limits prospects for agreement about best interests, at least at a policy level. Welie [76] notes aversion to the concept of intersubjectivity in the wider bioethics literature, suggesting more widespread belief in radical value pluralism.

Taken in sum, it is notable that there are a number of different strands of liberalism at work, which might place less [37, 76] or more [75, 87, 89] value upon subjective wellbeing and anti-paternalism. In one case at least [87], this subjectivism is radical enough to actively discount the possibility of intersubjective agreement at a policy level, dramatically limiting the role of the state. These views thus embrace not only different views of medical decision-making, but also of political form.

The first theme, then, sees a range of theories used to position, critique and explain best interests. The most prevalent inference is that best interests are consequentialist or utilitarian. The philosophical theories of prudential value are also linked to best interests. Best interests often have a legal character and brings Kantian concepts into discourse. The relationship to rights is prevalent, but views of best interests vary, with some rights bearers such as parents and people with disabilities being viewed as marginalised by the concept of best interests, while children are viewed as having rights compatible with best interests. Sources also drew on strands of predominantly liberal political philosophy which differed in the value they placed on subjectivism and anti-paternalism. Prima facie, a putative concept of best interests appears consequentialist in philosophy, legal in character, and paternalistic in creed. Yet this outward picture will be challenged as the themes proceed. In particular, the extent to which subjectivist and anti-paternalistic strands of liberalism shape best interests to exclusively focus on refusals (i.e. negative preferences) will become apparent in the next theme.

### Theme 2: Processes and factors for deciding for others

Medical decision-making processes are focused upon the autonomous preferences of the patient, but where the subject of a decision lacks capacity or competence, it becomes difficult or even impossible to access the patient’s first-hand account of their preferences. Accounts of best interests remained focused on these areas, and few examined the role of best interests restricting patient demands for treatment.

#### A typology of processes

**Fig. 1:**
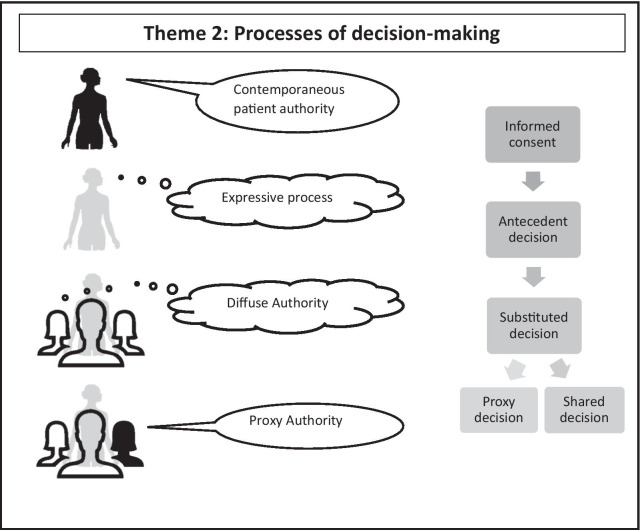
Processes of decision-making

Accounts of healthcare decision-making describe a variety of different process that can be used when a contemporaneous informed consent is impossible. Interpretations of best interests vary between sources, and it is ambiguous where ‘best interests decision-making’ begins. While various processes are described, a broad typology of three distinct decision-making approaches can be identified in addition to contemporaneous informed consent (Fig. 1). These are (1) ‘Expressive processes nominally giving a voice to the patient; (2) processes that ‘diffuse authority’ by sharing decision-making between multiple parties, and; (3) processes where ‘proxy authority’ confers authority on particular decision-makers.

*Expressive processes:* The informed, stable decision of the patient is widely agreed [48, 52, 60, 76, 81, 91, 92] to be the superlative way of assessing what will make things go best for that patient. A hierarchy of decision-making procedures [60, 72, 76] orders the desirability of procedures according to their similarity to a contemporaneous expression of preference. An archetypal hierarchy [93] cited by several sources [48, 60, 76] ranks informed consent the ideal decision-making form, followed by an advance (antecedent) decision, and a substituted judgment—where “the decision maker makes his or her decision on the basis of what the mentally incompetent person would have done if s/he had been competent” [53]—the third. Most distant from the ideal is ‘best interests’.

Despite this hierarchy, the relationship between best interests and these ‘expressive’ processes is ambiguous. Best interests and antecedent decisions are sometime held to overlap [72] because both centre on ‘critical interests’—the enduring lifelong interests of continuous individuals [94]. Others [54] argue the opposite: best interests ignore the ‘critical interests’ expressed in an antecedent decision, and instead focus on “objective” and generic factors.^4^ Some suggest the division between best interests and other processes is one of the presence or lack of explicit authorisation [89]. Again, this suggests that substituted judgment is a form of best interests, a position to which English law is seemingly amenable [75]. Such a view is reinforced by criticism of the putative basis of substituted judgment in autonomy [95] especially where a patient’s desires have never been known [46, 48, 53, 55, 69, 76, 86, 88, 96]. Overall, the tone of the literature reviewed indicates ebbing confidence in expressive decision-making as the process becomes more distant from direct self-expression.

*Processes that diffuse authority:* Where expressive process cannot be used, the decision-making authority is transferred elsewhere. Decision-making processes that diffuse authority (that is, draw upon a variety of viewpoints, none of which is singularly authoritative) are advanced by several authors [44, 46, 48, 50, 54, 59, 84]. Occasionally these accounts are linked to wider theories of shared decision-making [85] or intersubjectivity [76].

*Processes that reserve proxy authority:* Although processes that diffuse authority are generally endorsed, who should be included in the decision-making group, and how this authority should be apportioned, varies. In adults, High [48] argues that clinicians must scrutinise surrogate decisions to make sure they are in the patient’s interests, while Taylor [75] argues this is defacto the case in English law since it is only after clinicians have determined treatment that the question of whether the treatment is in the patient’s best interests can be decided. Certainly not all preferences are respected. Although more rarely discussed, both patient [95] and proxy [67] demands for treatment are restricted by ‘objective’ interests [68]. Most commentators suggest these restrictions consist of medical opinion of benefit or interests [42, 74, 76] and consideration of resources [95]. Nevertheless Snelling [77] asserts both a more protective function in restricting proxy abuses of power or discriminatory values, acknowledging moreover that such abuses might arise from failures in social support of families. This notwithstanding, the picture of preference is clearly revealed as one that protects negative rather than positive preferences.

In decisions about children, it is common to assert that parents should exercise prima facie authority [34, 59]. Others indicate a process where parents are given the ‘facts’ by a variety of expert parties, from which parents then choose according to their ‘values’ [44, 85]. Others harness the fact/value distinction to assert certain parties have privileged access to the patient’s ‘values’: these include neonatal nurses [97] and adult patients’ closest relatives [52]. It is notable that what distinguishes a ‘value’ from any other preference (say, an aesthetic one) remains ambiguous. Processes that reserve proxy authority are assumed to identify the proxy who might best approximate the patient’s preferences or interests. Arguments that proxies may decide on the basis of reasons independent of the patient’s viewpoint is discussed in theme 3.

A summary of the various process described in this typology can be represented diagrammatically (see Fig. 1).

The typology indicates that the goal of satisfying patient preference—and thereby putatively to allow patients to express their values—motivates decision-making processes. It also shows that innovations to liberalise decision-making for others have led to a particular emphasis on negative preference satisfaction in best interests, with positive interests at best vaguely identified with medical duties, suggesting that forms of liberalism that value subjectivism and anti-paternalism are ascendent. Where knowledge of a patient’s preferences become weak or absent, decisions move into the realm of diffuse authority and become shared decisions or proxy authority to become proxy decisions. Here, the emphasis on preferences in best interests means that such decisions may paradoxically be guided by the principle of preference satisfaction, but given the inaccessibility of actual preferences, other interests must be identified to share the work. The literature identifies a number of such factors which we can explore.

#### Factors

Given the dominant aim of preference satisfaction, the leading factor identified by the literature is patient preferences. Underlining the ambiguous nature of best interests, studies take two different approaches to the relationship of best interests and preferences. Some identify preferences with best interests, arguing that best interests are the goal of every decision made about the patient’s healthcare including decisions made by the patient [52, 77]. These accounts take the view that best interests must reflect the patient’s preferences whatever else they reflect. Others acknowledge that goods besides preferences may be interests [38] but that only a preference can decide best interests. Against these accounts many sources separate best interests from preferences [34, 37, 47, 67, 78, 84, 87] (or choices [63, 64, 67, 83]) arguing that best interests is suited to those whose own decisions are unascertainable. Thus we have two distinct accounts of best interests. On one, best interests occupies a distinct area that is apart from preferences (even if a decision may also take account of preferences). On the other account, best interests are essentially indistinguishable from preferences.

Besides preference, many accounts offer other factors that may putatively inform decision-makers. Often accounts give minimal detail of these factors. A sense of their range is provided in Table 1.

**Table 1:** Factors in best interests decisions

Factor	Sources and notes
Conscious experience	Conscious experience is defined as sentience [55] which manifests (at minimum) in short-term recall [63]. It is suggested as a factor by a number of sources [50, 57, 61, 63, 92]
Dignity	Identified without definition by [49, 60, 62, 86, 92]. Defined as inalienable rights to equal treatment and respect [61, 87] and a recognition of common humanity linked to human rights [55, 77]
Medical interests	Clinical needs/medical interests are identified as factors in best interest [75, 76]. Identified as a potential narrative of best interests offered by the courts [75]. No specific definition of what counts as a medical interest
Benefits and burdens	Many sources identify benefits and burdens [44, 46–55, 58–62], with a variety of examples given
Pleasure and pain	Pleasure exclusively identified as an interest of children [34, 56]. Pain Identified as pain [83] or harm [86]. Some [57] argue pain is only against interests to the extent a person is aware of it
Quality of life	Frequently identified [49, 50, 52, 54, 55, 57, 60, 62, 85, 88, 98]. Defined as either subjective or objective value of life [49, 54, 73, 75] Authors argue that quality of life is an implicit factor guiding legal [49, 62, 98] and clinical [44, 50] best interests decisions or used as explicit cover for arbitrary and prejudiced decisions [44, 52–54]
Futility	Identified by [49, 53, 61, 76, 88, 98, 99]. Sometimes [44, 62, 75] identified with a medical judgment
Effective treatment possibilities and prognosis	Identified by [44, 86]
Developmental potential	Identified by [34, 50]
Medical progress	Identified by [51] in context of research
Sanctity of life	Noted in the context of law [44, 54, 55, 59, 95]. Defined as the inviolability of life [44]

A scoping review cannot provide a comprehensive account of all factors that could be cited in discussion of best interests decisions. Even accepting this, the factors captured in Table 1 are often advanced with little definition or analysis: in fact, only a handful of sources [61, 63, 73, 77, 87, 92] embark on detailed discussion of factors they name.

There are potentially several reasons for the lack of definition. Some factors may appear self-evident, for example pleasure and pain. Moreover, the need for brevity in academic publishing means that detailed discussion of all terms is no more practical in these sources than in this article. This notwithstanding, many of the factors appear to be part of circular definitions that do little to shed light on the concept of best interests: for example, quality of life is sometimes used to define best interests in a way that does not close the question of what either concept means. Some of the sources attack these factors for just this reason, but enough use them in this way to suggest this open position is intended. To define factors such as e.g. futility or dignity is to subject them to external evaluation. Failing to define futility or dignity invites others to do so, either though proxy authority or within diffuse authority. Thus these factors are to be finessed privately, not signal explicit approaches. In this way, under-definition can also be seen in the light of those liberal theories that emphasise subjective evaluation and anti-paternalism rather than positive rights, interests or welfare. The strategy is also compatible with an orientation toward personal preference because factors like quality of life, futility and dignity, when subjectivised, conflate with preferences. On the other hand, this means that a vacuum left by under-definition pushes best interests accounts ever further toward reduction to individual refusals. In this way liberal anti-paternalism also acts as a brake on the scope of any detailed factors beyond a ‘negative preference’ account. It thus limits the scope of best interests judgements. This could be unhelpful, most obviously where preferences are not accessible.

It is unclear if this is a conscious strategy. Given that the bulk of commentators viewed best interests as distinct from preferences, the lack of serious attention to the question of what should inform best interests decisions seems curiously myopic. Where there is not simply an assumption that some preferences can be divined and/or that a proxy is authoritative (see theme 3, below), this myopia may perhaps be explained if it arises from tacit acceptance that what is best is largely a matter of (inter)personal negotiation and discretion among decision-maker(s), indeed, this may be why there is so little influence of the accounts of positive rights that are asserted in the cases of children and persons with disabilities. Thus it may simply be that best interests, in the context of diffuse authority, is a matter of working out differences between decision-maker preferences, rather than any more principled approach that might assert more generally applicable positive interests. I will consider further what this says about best interests in due course. For now, I will consider the final theme. This details attempts to bypass the problems of finding the patient’s preferences by taking a relational approach to best interests decisions.

### Theme 3: Best interests and the challenge of relationality

Best interests is often noted to take a patient centred approach—the interests considered apply to the patient rather than to society or some other person. This is congruent with the focus on liberal rights noted in earlier themes. Nevertheless a challenge to patient-centred best interests is that the perspective overlooks the legitimate interests of other parties. A number of sources offer alternative, relational, perspectives by re-conceptualising interests as socially connected. The gap between patient-centred and relational accounts may nevertheless remain blurred: many relational arguments suggest relatives have the best understanding of patient preferences. Despite this, two distinctive, non-patient-centred arguments are present in the literature. Firstly, persons other than the patient should be prioritised on the basis of their comparatively greater interests. Secondly, some sources prioritise family interests over children’s interests on the basis of the special nature of childhood.

#### Against patient-centredness

Best interests is often identified as a standard that is focused on the individual [46, 50, 56, 57, 65, 69, 70, 75, 77, 86, 87, 92, 98]. A patient-centred perspective is argued to be protective of a patient’s reflective values [95] and to appropriately focus doctor’s duties [47, 88]. The focus on the individual predominates to the extent that self-interested explanations are advanced for activities that are—prima facie—other-regarding, including non-therapeutic research in children [51] and organ donation [67, 99].^5^ However, the failure to adequately consider the interests of persons other than the patient is allegedly a defect of the best interests standard [42, 47, 57, 68, 70].

Three arguments against patient-centred best interests can be identified. Firstly, if all interests are equal,^6^ focus on an adult or child patient’s individualistic best interests gives unfair priority to that patient’s interests in the face of strong countervailing interests of others [42, 47, 70, 98, 100]. Second, a web of interconnected interests ties patients to their communities or families, such that it is impossible to identify individual interests. In the case of children, the distinctive, close relationship of parent and child intrinsically counts against viewing children’s interests individually [56, 59, 65, 68, 77, 78, 83]. In adults, community beliefs and interests frame the patient’s best interests, either because individual interests may align with those of a wider group [66] or because the individual interests may be moot without a community to enable them [87]. Third, some assert that decisions detrimental to a child’s individual wellbeing may sometimes be valid because the family itself has ‘group’ interests that have prerogative over the claims of individual family members [56, 68, 86].

All three arguments are primarily challenged on an empirical basis. Legal commentators note best interests judgements appear sensitive to impacts on both family and public [42, 44, 49, 69, 84]. In decision-making at large, others note that, realistically, decision-makers may privately include such considerations in any event [58, 92]—although for some this is proof of the opacity that makes the best interests standard defective [57, 70]. A number of mechanisms within the rubric of best interests appear to recognise the interests of others: strong rights of parental discretion in many (if not all) jurisdictions utilising best interests [69]; that best interests is recognised as only ‘*a* primary’ consideration (opening the door to a range of competing ‘primary’ considerations) in the UNCRC [78]. Others argue that most commentators recognise that the child’s best interests is bound up with the wellbeing of their parents and siblings [34, 68], thus it is not clear that a patient-centred standard fails in the way critics claim.

#### Family self-determination and parental autonomy

Several commentators argue that family involvement in decisions gives insight into the preferences and values of the adult patients [52, 62]. Others argue that, because family decision-makers are preferred by patients themselves, there must be a rebuttable presumption that families are acting in the patient’s best interests [48, 75]. Only one source suggests that the role of families in adult decision-making should echo those of authoritative parents in decisions about children, arguing that families should have rights of self-determination as the key social unit of society [48]. Although there may be a tendency for clinicians to defer to family wishes in any event [74], most commentators remain cautious about the reliability of intra-family understanding of one other’s desires [64, 75, 76].

The story is rather different for children, whose position is most in line with the relational view on best interests. Although ‘children’ encompass a range of ages and competencies, with rare exceptions [37, 77] the literature considers children at the lower age range. Here children are sometimes asserted to inherently lack qualities such as rational thought that underpins concepts of autonomous reasoning [46, 51, 86].^7^ Discourse around children’s best interests tends to recognise and endorse parental discretion on the prima-facie presumption that parents act in their child’s best interests [44, 51, 56, 58, 65, 67, 69, 85]. Some authors assert that this presumption is underpinned by autonomy, explicitly identifying parental discretion as a parental ‘autonomy’ right to exercise their values [44, 85]. On this view a parent with a valid, intimate, connection to the child may exert autonomy, either on the child’s behalf [44, 86] or to advance their own interests [85].

As we have seen, the relational critique can be broadly challenged by assertions that the version of best interests it attacks is a straw-man that is not congruent with practice. Yet it is notable that the defence relies on conceding that interests are already recognised as relational and interconnected. The case for relational best interests is most strongly asserted in the case of children. Here, the near-consensus on parental discretion lends assertions of parental autonomy coherence, despite the dominance of patient centred positions in the wider discourse. While the position of adults is more nuanced, it has apparent utility in solving the hardest problems of preference evaluation. Given that the process of shared decision making is already bifurcated between diffuse authority and proxy authority, it is apparent that assessments of best interests may move from a recognition of a *privileged* epistemic viewpoint of family members to an *authoritative* epistemic viewpoint. As I shall discuss in the final section of this article, the shape of best interest in future policy will depend in a large part on how this is held coherent with dominant strands of liberal thought.

## Discussion

The first theme indicates the broad structure of bioethical theory that was discussed in relation to best interests decision-making. There we see a variable relationship between best interests and rights, with favourable views of best interests within children’s rights discourse, but critical views in discourse on disability rights and parental rights. Best interests is both a bioethical concept and a legal concept, hence, despite being held to be consequentialist or utilitarian, best interests discourse looks to specific deontological concepts and rules. Best interests is identified with prudential value theory, which concerns what is good from the point of view of the individual, yet distinct strands within this theory are judged to be inadequate to inform the practicalities of best interests decision-making. Finally, best interests is identified with liberal political philosophy, and many of the ideas within best interests discourse are implicitly grounded in commitments to various strands of political liberalism.

The effect of liberal philosophy on best interests may be reductive, for in the second theme only a narrow spectrum of liberal thought, that emphasises radical subjectivism and anti-paternalism, is discernable. This leads to an emphasis on preferences that both influences and limits interpretations of best interests. The *Influence* of the preference emphasis is seen in significant innovations in best interests decision-making. These efface best interests to the extent that where best interests decision making begins is ambiguous—it could be once contemporaneous consent is exhausted, but could just as easily be where (in my typology) expressive decisions yield to diffuse or proxy authority. Influence is also seen in the typology of processes of decision-making for others. Here processes are commonly ranked according to their distance from preference, and best interests viewed as a process to be undertaken only if unavoidable. As patient preferences become impossible to replicate, decision-making authority is diffused among a wider group in a manner akin to shared decision-making. At this point the process pathway bifurcates either into a process without a specific identified authority, or a process where a specific proxy is given an area of authority. Few explicit factors to guide the latter models of decision were identified. The theme further shows that these forms of liberalism *limit* best interests decision-making in that the diffuse authority models are largely devoid of guiding factors that can operate without direction from preferences. Moreover, restrictions on requests for treatment throughout show a strong bias toward negative, rather than positive, interests.

The final theme explores relationality, where those who have a pre-existing relationship with the patient take an increased role and status in decision-making. Advanced as an alternative to the patient-centred approach, relational approaches are often justified on the basis that persons are interdependent. Any decision thus affects a network of legitimate interests that must be accounted for. While logically this may imply strong disbenefits to the patient, relationality is nevertheless often aligned with patient-centred approaches by arguing that those in existing relationships know the patient best (so can approximate the patient’s preference best) and can be relied upon to put the patient’s wishes (or some other form of wellbeing) first. In the case of children, many commentators suggest relational decisions by parents should be subject to an adequacy threshold. From the perspective of the literature, relationality appears the only candidate theory advanced to fill gaps left by both a focus on negative preferences and under-theorisation of factors that we have seen in theme 2.

We can make further sense of the bioethical approach to best interests by comparing it to the way best interests occurs in wider historical and current non-medical discourse. Recall that my discussion of these areas suggested that best interests involved certain actors, approaches and ends. The theoretical discussion in the bioethics literature captured in theme one indicates a fairly balanced approach to these areas, with theoretical attention to both citizen and state actors, approaches to positive and negative rights and/or interests, and the potential for both marginalisation and protection. However, the bioethical innovations in processes and mapping of factors in theme 2 reveal a more lopsided focus. Attention to diffuse and proxy authority is cognisant of the various actors in best interests decisions, and the emphasis on preference indicates an awareness of, and resistance to, the potentially marginalising function of best interests. However, the reductive approach to factors suggests an inattention to positive interests which are, at best, vaguely characterised as medical duties. This is compounded by rather limited discussion of restrictions on patient demands for treatment, despite these restrictions presumably themselves implying a level of paternalism. Altogether, this reveals a striking lack of attention to positive interests. The final theme again attends to the actors as well as, potentially, ends of best interests, by the introduction of an adequacy threshold. While in theory a threshold suggests that there is scope for attention to positive interests, the dominant harm threshold once again focuses on negative interests, both of the parents or family to a zone of non-interference from the state, and to the child or patient in terms of specifying prohibitions rather than duties on parents or the state.^8^

As we noted at the beginning of this article, critics of best interests in bioethics tend to focus on the vagueness of best interests and its potential to be used by the state to covertly marginalise and restrict the liberties of persons with disabilities and parents. Defenders assert its protective potential and discretionary advantages. Debates on vagueness and marginalisation suggest identification and focus of both positive and negative interests should be present in the literature, yet taken as a whole there is a more sustained emphasis in the literature on negative interests, especially when the practicalities of best interests decisions are discussed. The literature does contain some implicit, and more rarely explicit, identification of positive interests—for example in identifying certain positive medical duties or in papers endorsing children’s rights. These are not operationalised or clarified. It is possible that some focus on positive interests is obscured by a sense that rights and positive interests will be operationalised in private interactions at a patient level. Yet what is notable is the lines of debate: positive interests are not generalised, and broader accounts of positive interests are either defended or rejected wholesale. It is only negative interests that are open to debate, generalisation or limitation.

Returning to the theoretical underpinnings identified in the first theme I will argue that these blind spots and under-theorisations arise from broader political, rather than moral or empirical, viewpoints. A focus on negative interests reflects background assumptions during an era of radical retrenchment of the welfare state. Through such a frame the role of relationality becomes one of shaping best interests into a form more fitting to the times.

### Theorisation of best interests: explaining negative interests

Best interests is a broad and amorphous concept, that fulfils numerous functions in the decision-making environment [105]. As Bester [34] notes, best interests sometimes receives apparently wilfully hostile interpretation from its bioethical critics. Bioethical critiques generally focus on trying to combat the marginalising potential of best interests on persons with disability and parents. Herein they address the vagueness of best interests by defining—or contesting—areas of negative interests. Yet these debates overlook the fact that the boundaries of this debate are defined in line with changing political beliefs. Ultimately by shaping the questions that bioethicists seek to answer, these beliefs appear to be surer indicators of the way best interests may evolve if it remains at the centre of medical ethics and law. I shall now argue that neither the ethical framework of consequentialism, nor the empiricism of value theory can provide convincing explanations for the focus on negative interests in shaping best interests as a concept. Deontology has more explanatory power, especially in view of interpretations of Kant that are seminal to bioethical discourse. Such interpretations, in turn, speak to political philosophies of liberalism that shape medical law, yet these philosophies are not static and the development of bioethics has tracked radical changes of the dominant liberal philosophy in wider politics and economics.

I start by considering the claim that best interests is consequentialist. Although frequently repeated in the literature, this claim is rarely accompanied by sustained reflection, and appears weak when examined. Labelling best interests as consequentialist simply because it pays attention to consequences is unconvincing, perhaps arising from an oversimplification of deontology as unconcerned with consequences. This is clearly incorrect: to take Kant’s first formulation of the categorical imperative as an example, imagining a maxim as a universal law of nature implies an antecedent assessment of consequences [106].^9^ More credibly, utilitarianism may follow from Buchanan and Brock’s claim that, after consideration of all interests, best interests instructs that the “course of action to be followed … is the one with the greatest net benefit to the patient.” [93]. This could imply act-utilitarianism (where every action is calculated solely to maximise utility production), although this makes assumptions about the claim, which does not specify a principle of utility. Potentially a focus on maximising health without respecting any other value could be psychologically true if decision makers follow a single heuristic [108] (like health) as a proxy of best interests. However, it seems unlikely that any well-intentioned decision-maker would consciously avoid the ‘best’ outcome overall for the patient all things considered, and this could as easily be described by a deontological rule as utilitarianism. Thus there is no strong sense that consequentialism underpins the way best interests are theorised in bioethics.

Parallels between best interests and prudential value theory have long been drawn [71]. Unlike consequentialism, prudential value theory could suggest potentially strong underpinning of preferences in best interests through desire theories of value. Innovations such as antecedent decision-making and (more controversially) substituted judgment have created a framework for assessing preferences even where a patient lacks the ability to express a contemporaneous—or perhaps even any advance—preference. However, these innovations lack empirical teeth because they do not make it immediately clear *why* it is better to look so hard for preferences in these circumstances. Prudential value theory, in advancing ideas of what is ultimately good for people, is a potential way to add strength to these accounts. Yet the desire theory of value unfortunately contains stumbling blocks to this strong account. For respect for preferences to be underwritten by the desire theory of value, preferences must be well-informed, well-motivated and be made by actors who will be aware of the outcome. Desire theory is therefore a poor empirical fit to many everyday cases of decision-making [109], and even more so once decisions cease to be contemporaneous [110]. More broadly, there remain great difficulties finding a theory of value that is both normatively and empirically successful [111]. This makes eclectic use of different branches of value theory seems warranted [72, 73], although explanatory success may also be found with more novel approaches such as eudaimonism [112] or perfectionism [113]. Nevertheless, although these novel theories should therefore not be discounted, they may either be reduced to preference accounts, or be too strongly deterministic to be normatively accepted as a priori guides to best interests decision making.

It may seem that deontology has similarly bleak prospects of furnishing an account that can underpin the bioethical approach to preferences, since the primary exponent of deontology—Kant—is often held to be stringently rationalistic [114]. Yet the capacity of Kantian autonomy to underwrite preferences strongly depends upon the account of Kant that is followed. The seminal bioethical understanding of autonomy promulgated in Beauchamp and Childress’ [45] principlist approach proposes a low bar to judging a preference ‘autonomous’. The approach has been criticised [115, 116] for allowing immoral and irrational actions and thus conforming, not to a Kantian view of autonomy, but ‘heteronomy’ (i.e. action based on causes other than self-legislation of the moral law). Nevertheless, by adopting a narrow view of rationality, this critique ignores the fundamentally politically liberal intent that can be attributed to Kant. The constructivist view of Kant championed by Rawls [117]—and likely influential on Beauchamp and Childress given the account’s proximity to their seminal 1979 work—argues Kant’s broader intent is to identify autonomy as the key factor in the process of personally constructing a view of the good.^10^ Such a view informs a politically liberal view of a world characterised by the liberty and equality of autonomous citizens [119].^11^ The theory of autonomy is most distinguishable in this modern Kantian form, where not only is it held to inform the theories of rights we have encountered, but also the post-war development of rights theory [122]. Thus it is the normative philosophical basis of liberalism alone that cashes-out preference in a world of medicine without medical authority. Yet this elevation of preference does not fully explain the orientation toward negative preferences defended in bioethical discourse.

Since numerous duties relating to best interests are framed by legal discourse, it is in law that we see this form of the liberal basis of best interest take further shape. This appears most true for discussion of rights, where the legal status of the UNCRC and the UNCRPD means that best interests and rights have radically different affinities and conflicts. The UN committee and some supporters of the UNCRPD have argued that best interests signals the legal inequality of persons with disability [19], whereas the prominence of best interests in the UNCRC means that children’s rights discourse often strongly defends the concept of best interests. Again, the dichotomous views of disability and children’s rights discourse are the result of the (un)acceptability of best interests as a frame for generalised goods, but both assert some positive rights. Parental rights discourse, less closely associated with law, stands apart these debates inasmuch as it appears more concerned with asserting parental authority over children [99]. At least as parental rights are asserted in the bioethics discourse, it seems most amenable to a focus on negative rights.

This association of bioethics with negative interests seems at least partly explicable by considering political changes unfolding at a time when academic medical ethics was developing. Early bioethical critiques arose at a time when ideas of positive interests encapsulated in the growth of post war welfare states were falling from political favour [123]. While ethical critiques from law argued that best interests was a front for closed medical authority over patients [124], the increasing attention of the law to medical decision-making was itself part of a reaction to fundamental shifts in the political zeitgeist. Here, emphasis on personal responsibility went hand in hand with radical welfare reform. These changes moved healthcare from a realm protected from the vicissitudes of the market, to the realm of contractual bargaining [125]. The shift to contractualism in turn has been argued to have led to increasing recourse to tort law to redress the deficits of a fragile state safety net [126], which placed best interests further under the legal microscope. While many early bioethicists identified with political liberalism [127], liberal theory was itself changing as previously less influential strands became dominant.

### The changing vogues of political liberalism and the development of best interests decision-making

Liberal philosophy is dominant, if implicit and unquestioned in most accounts seen in this review: indeed, the fractured relationship of best interests and rights bears out this implicit liberalism, given that the liberal rights of children and families [128, 129] and people with disabilities [93, 130, 131] are areas of difficulty for most forms of liberal philosophy. Some accounts characterise the implicit liberalism of the theoretical landscape of healthcare decision-making as largely monolithic [114], yet my analysis suggests this presumption requires challenge. Liberalism includes a huge latitude of thought, ranging from Hobbes, to Thomas Hill Green, to James Buchanan [132]. The liberal political philosophy identified by the literature is that of Mill, Rawls and Berlin—all of which are broadly compatible with the ‘new liberal’ tradition of Green and Dewey that influenced the growth of post-war welfare states.^12^ Nevertheless, the literature also draws attention to the impact of libertarianism—more familiar in European terms as ‘neo-liberalism’ [134]—on the wider political economy. As noted above, seminal bioethical theory has debts to Rawls that gravitate toward 'new liberalism'. Yet while Rawls was able to accommodate protections of equal liberties and redistribution of social goods within his view of pluralism [90], the emphasis on negative interests (and the failure to coherently articulate positive interests) in best interests discourse suggests that bioethics struggles with attempts to accommodate value pluralism by agreeing common goods. Indeed, once the bioethical commitment to value pluralism becomes radical, it becomes incompatible with positive interests altogether, because the diversity of values means there is no possibility of agreeing the sorts of positive interests a society might assert. Such a view resonates with that of neo-liberal thinkers. While much of the work in the neo-liberal tradition is most familiar in economics, its political and normative implications have been examined in a recent monograph by Biebricher [134]. This analysis signals the important role of belief in radical value pluralism in the political views of influential economists such as Hayek, Rüstow, Röpke and Buchanan. Here, differences in individual preference preclude intersubjective agreement, and thus are best expressed by the operations of a free market because “a dollar vote is never overruled” [135](339). The positive role of the state is thus no more or less than to ensure that the operations of the free market are protected, since the market is the most competent guarantor of personal liberties.

Given the continued influence of these ideas in politics and policy, it is likely that they will continue to influence theories of best interests. The bifurcation between proxy authority and diffuse authority approaches to shared decision-making readily suggests tensions between new liberal and neo-liberal approaches. Limited possibilities for agreement disrupt or wholly undermine models of decision-making process that cultivate intersubjective agreement, such as those favoured by new liberals like Habermas and Rawls. Exponents of new liberalism have favoured inclusive expressions of popular sovereignty, typically following the Habermasian tradition of developing processes that diffuse authority to pools of stakeholders who seek consensus by “decentering” [136](77), but this is an arguably technocratic solution that can become mired in lack of agreement. Many neo-liberal thinkers have sought to remove moral decisions from a public sphere since “[m]oral responsibility is a person matter, not a social matter” [137](106). In her analysis of connections between neo-liberal economics and family policy, Cooper suggests prominent neo-liberals such as Becker and Friedman have seen the family “as the primary source of economic security and a comprehensive alternative to the welfare state." [123](9). Giving responsibility to the family in this way may offer decisiveness, albeit with no moral limits beyond the values of the family members operating in the constraints of the market.

## The rise of relational approaches

While a libertarian/neo-liberal view need not be explicitly endorsed by those with commitments to familialism or increased parental rights,^13^ it may well influence the sorts of assumptions that motivate the relational approaches seen in this review. Certainly the review indicates that theorists may turn to relational approaches to smooth over the difficulties that the liberalising innovations (discussed in theme 2) have in replicating preference for some patients. A relational approach may bypass these problems by sanctioning the preference of another decision-maker who is presumed to have the patient’s welfare at heart. While such an approach cannot per se amount to a *complete* solution to best interests decision-making, given the frequency of patients with no family or friends [138], yet it has clear merits both identified within, and beyond, those espoused in neo-liberal approaches: it coheres with common presumptions about the moral fitness of parental decision-making [139] and the desire of many adult patients to make decisions in consultation with their families [140, 141]. Moreover, it is simple and presumptively coherent with consent based decision-making approaches as a whole. Nevertheless, because it negates all thought of a separation of interests, a straightforward substitution of patient preference for an empowered proxy has no safeguard against disregarding the interests of the patient by those in a family relationship. Although this may be seen as a virtue by some commentators [142, 143] such sentiments seem both strongly at odds with the root justifications of relational autonomy which build on the mutual social connections that make choices possible [144]. Indeed, Jennings [145] warns that relational autonomy should therefore “not be conflated with collectivism or with authoritarian and paternalistic versions of communitarianism”. Yet it is predominantly an authoritarian characterisation that emerges from my review.

One way of accommodating concerns that proxies may judge poorly is to identify when a threshold of acceptability is exceeded. This has been a popular approach in paediatrics and in particular interest has coalesced around the ‘harm threshold’ [146, 147]. The increasing scrutiny that has fallen upon the harm threshold as a result indicates ongoing problems with both the conceptualisation [148, 149] and the mutability [150] of that threshold. These criticisms seem to have purchase far beyond the harm threshold itself, raising the possibility of more general limitations to principles in providing decipherable boundaries of practice. Such limitations occur whatever approach is taken—authoritative proxies or diffuse authority, best interests or harm threshold. Nevertheless, avoiding a mere modus vivendi between competing decision-makers or unaccountable authority on the part of an authoritative proxy suggest some guiding factors or positive interests are needed to lead decision makers to a principled decision. As I have noted, while factors appear, these concepts can themselves be cyphers for the dominance of agent-preference based accounts [151], leading to a circular train of reasoning. Meanwhile positive interests may be contained in existing rights but are rarely further articulated, or within vague notions of medical benefit that are simply inadequate to indicate which of the plurality of positive goals of medicine are ethically acceptable. A failure to publicly articulate positive interests could mean there is no map showing where a proxy or diffuse authority process should aim. One task of ethics should be to articulate the good. In this debate at least, bioethics appears to have abdicated this role, occupying a position where debate centres on negative interests.

If, as seems likely, it is the background socio-political conditions that define best interests decision-making, what appears urgent is to understand how these conditions influence practice, both when they cohere with popular sentiment and when they present practice with insuperable difficulties. Of the latter, certainly concepts like personal preference must be problematic anywhere where there is no personal preference to be found, since inconsistencies may lead to a gap between public conduct and private practice. Reasons to act may be found, based on private evaluations of positive interests, but deviance from legally or ethically endorsed approaches may inhibit discussion of dissonant realities, meaning that the norms guiding practice are largely unarticulated. This, of course means that *how* decision-making takes place in practice deserves careful and detailed empirical investigation, including the way decision-makers navigate the dense webs of family relationships and express the theories that have shaped ‘best interests’ in bioethics.

## Conclusion

This scoping review sought meanings and theorisations of best interests in 57 papers. The three themes offer accounts of (1) theories that border or underlie best interests; (2) the processes involved in decision-making for others and the factors that inform them, and; (3) the role of relational factors in best interests decisions. The theories cited span consequentialism, value theory, deontology, rights and political philosophy. Nevertheless, consequentialism fails to give a descriptively strong account of best interests, while value theory, even in the preference-orientated guise of desire theory, fails to provide a credible empirical and normative grounding of preferences, especially of those whose preferences are unknown. Analysis of deontology and law point toward a dominant role of liberal political philosophy in formulating the discourse around decision-making. Processes and factors are strongly preference based, but limited to refusals rather than to requests, suggesting a tendency toward a liberalism that emphasises negative interests. As difficulties in accessing preferences mount, processes shade into either diffuse authority, where authority is shared between decision-makers, or proxy authority, where an empowered proxy made decisions on the patient’s behalf. What informs these processes seems largely private and discretionary, since the few named factors that guide best interests decisions tend to be sparsely defined and easily beg the question of preferences. One way to make progress where preferences are absent is to enrol family members as decision makers in relational approaches. Solutions tend to emphasise proxy authority. Overall the literature strongly favours negative interests in formulating best interests, with some identification or implicit defence, but little debate, of positive interests or factors. While liberalism dominates discourse, bioethical theories of best interests span new liberalism and neo-liberalism. My analysis suggests neo-liberal philosophies that favour negative interests and private resolution are ascendant and have profoundly influenced the theorisation of best interests. Nevertheless is it unclear how this discourse is navigated in practice.

While questions remain, what is clear is that best interests does not neatly fit into the conceptual frames suggested by its detractors or defenders. It is neither clearly paternalistic or consequentialist, nor is it necessarily holistic and patient centred. The dominant role of neo-liberal philosophy in shaping the current best interests standard shows that best interests is informed by a range of socio-political concerns and conditions beyond normative ethics. This article has considered how these are negotiated in the viewpoints of the academy, yet how such conditions are negotiated in clinical practice remains a vital question if we are to develop effective and ethical policy.

## Supplementary Information

**Additional file 1.** Detailed Methods.

## Data Availability

The datasets used and/or analysed during the current study are available from the corresponding author on reasonable request.

## Abbreviations

UNCRC: United Nations Convention on the Rights of the Child
UNCRPD: United Nations Convention on the Rights of Persons with Disabilities

## Footnotes

1 The authors attribute definitions of these elements to Freeman, the framework could not be found in their citations. See: [79, 80]

2 McMath [81] draws this view from Dworkin’s theory that rights have priority over interests see: [82].

3 There is some ambiguity about the status of best interests in this process. Not all advocates of thresholds specify a decision-making goal of parental decision-making, and strictly speaking this could represent a two-step process for deciding when a best interests determination should take place, rather than a process for determining best interests in the round. However, in most cases where it is specified the goal of both parental and state decision-making is the best interests or welfare of the child (e.g. [44]) suggesting thresholds are an identifiable process.

4 There is nevertheless considerable confidence in antecedent decision-making. Indeed, so great is the confidence of some commentators that they favour a patient’s antecedent wishes over their current behaviours [54, 63, 75], although others argue this is a misapplication of autonomy [92].

5 These sorts of cases of (arguably) tenuous self-interest are subject to a criticism raised by Groll [100]. Groll claims that the dominant norm of focusing on the patient leads to claims made in the interests of others being translated into claims made on behalf of the patient. To the extent that the self-interested reasons given in these examples are tenuous, they seem to confirm Groll’s claim.

6 Brody and Bartholome [47] argue it is “arbitrary” (p38) to give priority to the interests of the patient, suggesting the inspiration in this case is a utilitarian inclination towards person-neutrality.

7 We note this philosophical account of adult and child reasoning is challenged by a range of scientific studies. See e.g. [101–103]

8 This is at least the explicit case. Some seminal accounts of the harm threshold in bioethics suggest harm incorporates “basic interests”, although the positive interests inherent in such a concept are neither elaborated nor suggested to be more than a minimal account of interests. see: [104]

9 We agree with Rawls that the briefest consideration of moral philosophy tells us that “[a]ll ethical doctrines worth our attention take consequences into account in judging rightness. One which did not would simply be irrational, crazy” [107].

10 Much of this approach turns on whether we translate Kant’s use of *vernünftig* as ‘reasonable’ or ‘rational’: in German it means both. See: [118].

11 Also consider [120] especially pp 218–219; Indeed, such a view seems more likely to ensure the survival of autonomy given the strong challenges to the Kantian model of *rational* legislation from moral psychology e.g. [121].

12 Berlin is probably the most ambiguous of these thinkers, identifying himself in 1954 as “on the extreme right-wing edge of the left-wing movement” [133](437).

13 Indeed, Cooper [123] notes that thinkers on the social democratic left may view the empowerment of the family as expression of Polyani’s ‘double movement’ where individual empowerment is a corrective to excessively control of the market. In the absence of strong positive rights for social support I suggest that the fragmented and fragile nature of family support mean it is not a robust replacement for a social safety net.
