# Supplementary material for: The theorisation of ‘best interests’ in bioethical accounts of decision-making
Source: BMC Med Ethics. 2021 Jun 1;22:68. doi: 10.1186/s12910-021-00636-0 (PMC8167991; doi:10.1186/s12910-021-00636-0)
Supplement: Supplementary file 1 — Additional file 1. Detailed Methods. [file 12910_2021_636_MOESM1_ESM.docx]

# Additional File 1 – Detailed Methods

### Research objectives and keywords

The research aimed to map theoretical understandings of best interests that inform arguments about best interests in bioethics. In order to satisfy this objective, the following research question was devised to guide the review process:

“How was best interests understood in arguments about the best interests standard in relation to medical treatment decisions found in theoretical studies?”

The research focus was on ‘best interests’ as a concept, about which I wished to access multiple theoretical framings. While aware from past experience of a vast literature on medical decision-making per se, I wished to filter out wider discussions, and actively rejected (potentially synonymous) terms such as ‘Welfare’, ‘Wellbeing’ and ‘Value theory’, which assumed specific theoretical backgrounds. Instead, I focused my search using a narrow set of keywords (table 1), connected by Boolean modifiers (AND/OR), and using wildcards (*) to capture wider uses of the root term. The Pubmed and JSTOR databases were searched. A further question was how to account for differences between national perspectives, given that best interests is a legal as well as an ethical concept that is important in many different common law jurisdictions. The decision was made to keep the focus international: there is much influence, particularly between English law and different sources of commonwealth law, but also US law in this area. Moreover bioethics is an international endeavour which claims to offer a generalisable account of ethical principles and theories that should influence healthcare delivery and policy, thus it did not seem justifiable to place national boundaries on the review beyond those already imposed by searching for English language texts.

The following limits were applied where the database allowed: – Since 1960, English language, Humans.

| **Search Keywords** | |
| --- | --- |
|  | “Best interests” |
| AND | Theor* OR idea OR model OR concept OR scheme OR thesis OR account OR reflection OR observation OR point of view OR critic* OR argument |
| AND | Health OR care OR medic* OR nurs* OR therapy |

*Table 1: Search Keywords*

### Screening

Articles were screened according to the criteria given in Table 2.

| **Screening criteria** | |
| --- | --- |
| Include | Papers, books, book chapters. |
|  | Articles that provide arguments for and against the best interests standard |
|  | Articles that consider adults and/or children, with or without mental capacity or maturity |
|  | Articles that consider healthcare decisions, including research |
| Exclude | Grey literature including theses, journalism, news reports. |
|  | Articles that do not make theoretical claims about best interests |
|  | Articles that do not critically engage, but merely mention or report best interests (e.g. presuppose the self-evidence of best interests) |

*Table 2: Screening Criteria*

The search results were imported into Endnote software with their sources carefully tracked. Duplicate papers were identified with the software and removed. References were screened by title into ‘include’, ‘exclude’ and ‘unsure’ groups based on the perceived relevance of the title of the paper to the areas of enquiry noted above. Two articles in the ‘unsure’ group which focused on social aspects of best interests were retained, as, on balance, they offered important context that may have been otherwise missing from the discussion. In total, 53 sources were identified from two databases. Despite experimenting with modified keywords, I was unable to capture all of the sources I considered relevant while still keeping results in manageable limits. Thus four additional studies that contained important theoretical discussions of best interests were added to the systematic results, bringing the total to 57 studies (Table 3).

Table 3: Articles reviewed

| 1. Allmark P, Mason S, Gill AB, Megone C. Is It in a Neonate's Best Interest to Enter a Randomised Controlled Trial? Journal of Medical Ethics. 2001;27(2):110-3. doi:10.1136/jme.27.2.110. |
| --- |
| 2. Archard D. Children, Adults, Best Interests and Rights. Medical law international. 2013;13(1):55-74. |
| 3. Bailey RS. In whose interests? The best interests principle under ethical scrutiny. Australian critical care : official journal of the Confederation of Australian Critical Care Nurses. 2001;14(4):161-4. |
| 4. Baines P. Family interests and medical decisions for children. Bioethics. 2017;31(8):599-607. doi:10.1111/bioe.12376. |
| 5. Berger JT. Is best interests a relevant decision making standard for enrolling non-capacitated subjects into clinical research? J Med Ethics. 2011;37(1):45-9. doi:10.1136/jme.2010.037515. |
| 6. Bester JC. The best interest standard and children: clarifying a concept and responding to its critics. J Med Ethics. 2019;45(2):117-24. doi:10.1136/medethics-2018-105036. |
| 7. Birchley G. What limits, if any, should be placed on a parent's right to consent and/or refuse to consent to medical treatment for their child? Nursing philosophy : an international journal for healthcare professionals. 2010;11(4):280-5. doi:10.1111/j.1466-769X.2010.00456.x. |
| 8. Brierley J, Shaw D. Premortem interventions in dying children to optimise organ donation: an ethical analysis. J Med Ethics. 2016;42(7):424-8. doi:10.1136/medethics-2015-103098. |
| 9. Brody H, Bartholome WG. In the best interests of. Hastings Cent Rep. 1988;18(6):37-40. doi:https://doi.org/10.2307/3563048. |
| 10. Cave E, Nottingham E. Who Knows Best (Interests)? The Case of Charlie Gard. Med Law Rev. 2018;26(3):500-13. doi:10.1093/medlaw/fwx060. |
| 11. Chan TK, Tipoe GL. The best interests of persistently vegetative patients: to die rather that to live? Journal of Medical Ethics. 2014;40(3):202-4. |
| 12. Coggon J. Mental Capacity Law, Autonomy, and best Interests: An Argument for Conceptual and Practical Clarity in the Court of Protection. Med Law Rev. 2016;24(3):396-414. doi:10.1093/medlaw/fww034. |
| 13. Cooper R, Koch KA. Neonatal and pediatric critical care: ethical decision making. Critical care clinics. 1996;12(1):149-64. |
| 14. Daniels KR, Blyth E, Hall D, Hanson KM. The best interests of the child in assisted human reproduction: The interplay between the state, professionals, and parents. Politics and the Life Sciences. 2000;19(1):33-44. doi:https://doi.org/10.1017/S0730938400008881. |
| 15. Dawson A. The determination of 'best interests' in relation to childhood vaccinations. Bioethics. 2005;19(2):188-205. |
| 16. DeGrazia D. Value theory and the best interests standard. Bioethics. 1995;9(1):50-61. doi:10.1111/j.1467-8519.1995.tb00300.x. |
| 17. Donnelly M. Decision-making for mentally incompetent people: the empty formula of best interests? Medicine and law. 2001;20(3):405-16. |
| 18. Dworkin R. Consent, representation, and proxy consent. In: Gaylin W, Macklin R, editors. Who Speaks for the Child? Hastings-on-Hudson, N.Y.: The Hastings Center; 1982. p. 191-208. |
| 19. Fenwick AJ. Applying Best Interests to Persistent Vegetative State: A Principled Distortion? Journal of Medical Ethics. 1998;24(2):86-92. |
| 20. Groll D. Four models of family interests. Pediatrics. 2014;134 Suppl 2:S81-6. doi:10.1542/peds.2014-1394C. |
| 21. Gutheil TG, Appelbaum PS. Substituted judgment: best interests in disguise. Hastings Cent Rep. 1983;13(3):8-11. |
| 22. Hall A. Making good choices: toward a theory of well-being in medicine. Theor Med Bioeth. 2016;37(5):383-400. doi:10.1007/s11017-016-9378-4. |
| 23. Halliday S, Witteck L. Decision-making at the end-of-life and the incompetent patient: a comparative approach. Medicine and law. 2003;22(3):533-42. |
| 24. Harvey M. Advance directives and the severely demented. J Med Philos. 2006;31(1):47-64. doi:10.1080/03605310500499195. |
| 25. Hester DM. Interests and neonates: there is more to the story than we explicitly acknowledge. Theor Med Bioeth. 2007;28(5):357-72. doi:10.1007/s11017-007-9048-7. |
| 26. Hester DM, Lew CD, Swota A. When Rights Just Won't Do: Ethical Considerations When Making Decisions for Severely Disabled Newborns. Perspectives in biology and medicine. 2016;58(3):322-7. doi:10.1353/pbm.2016.0004. |
| 27. High DM. Surrogate decision making. Who will make decisions for me when I can't? Clinics in geriatric medicine. 1994;10(3):445-62. doi:https://doi.org/10.1016/S0749-0690(18)30332-X. |
| 28. Hope T, Slowther A, Eccles J. Best interests, dementia and the Mental Capacity Act (2005). J Med Ethics. 2009;35(12):733-8. doi:10.1136/jme.2009.030783. |
| 29. Inwald D. The best interests test at the end of life on PICU: a plea for a family centred approach. Archives of disease in childhood. 2008;93(3):248-50. doi:10.1136/adc.2006.111120. |
| 30. Kadish SH. Letting Patients Die: Legal and Moral Reflections. California Law Review. 1992;80(4):857-88. doi:10.2307/3480699. |
| 31. Keating RF, Moss AH, Sorkin MI, Paris JJ. Stopping dialysis of an incompetent patient over the family's objection: is it ever ethical and legal? Journal of the American Society of Nephrology : JASN. 1994;4(11):1879-83. |
| 32. Kopelman LM. Using the Best Interests Standard to decide whether to test children for untreatable, late-onset genetic diseases. J Med Philos. 2007;32(4):375-94. doi:10.1080/03605310701515252. |
| 33. Lachance D. In re Grady: the mentally retarded individual's right to choose sterilization. American journal of law & medicine. 1981;6(4):559-90. |
| 34. Leuthner SR. Decisions regarding resuscitation of the extremely premature infant and models of best interest. Journal of perinatology : official journal of the California Perinatal Association. 2001;21(3):193-8. doi:10.1038/sj.jp.7200523. |
| 35. Lim CM, Dunn MC, Chin JJ. Clarifying the best interests standard: the elaborative and enumerative strategies in public policy-making. J Med Ethics. 2016;42(8):542-9. doi:10.1136/medethics-2016-103454. |
| 36. McDougall RJ, Notini L. Overriding parents' medical decisions for their children: a systematic review of normative literature. J Med Ethics. 2014;40(7):448-52. doi:10.1136/medethics-2013-101446. |
| 37. McGee AJ, White BP. Is providing elective ventilation in the best interests of potential donors? J Med Ethics. 2013;39(3):135-8. doi:10.1136/medethics-2012-100991. |
| 38. McGuinness S. Best interests and pragmatism. Health care analysis : HCA : journal of health philosophy and policy. 2008;16(3):208-18. doi:10.1007/s10728-008-0089-5. |
| 39. McMath A. Infant male circumcision and the autonomy of the child: two ethical questions. J Med Ethics. 2015;41(8):687-90. doi:10.1136/medethics-2014-102319. |
| 40. Mercurio MR. Parental authority, patient's best interest and refusal of resuscitation at borderline gestational age. Journal of perinatology : official journal of the California Perinatal Association. 2006;26(8):452-7. doi:10.1038/sj.jp.7211547. |
| 41. Morris MC. Pediatric participation in non-therapeutic research. The Journal of law, medicine & ethics : a journal of the American Society of Law, Medicine & Ethics. 2012;40(3):665-72. doi:10.1111/j.1748-720X.2012.00697.x. |
| 42. Prendergast TJ. Resolving conflicts surrounding end-of-life care. New horizons (Baltimore, Md). 1997;5(1):62-71. |
| 43. Raines D. Deciding what to do when the patient can't speak: a preliminary analysis of an ethnographic study of professional nurses in the neonatal intensive care unit. Neonatal network : NN. 1993;12(6):43-8. |
| 44. Rivers D. "in the Best Interests of the Child": Lesbian and Gay Parenting Custody Cases, 1967-1985. Journal of Social History. 2010;43(4):917-+. doi:DOI 10.1353/jsh.0.0355. |
| 45. Rose P. Best interests: a concept analysis and its implications for ethical decision-making in nursing. Nursing ethics. 1995;2(2):149-60. doi:10.1177/096973309500200207. |
| 46. Samanta A, Samanta J. Advance directives, best interests and clinical judgement: shifting sands at the end of life. Clinical medicine (London, England). 2006;6(3):274-8. |
| 47. Shewchuk TR. The uncertain 'best interests' of neonates: decision making in the neonatal intensive care unit. Medicine and law. 1995;14(5-6):331-58. |
| 48. Snelling J. Minors and Contested Medical-Surgical Treatment. Cambridge quarterly of healthcare ethics : CQ : the international journal of healthcare ethics committees. 2016;25(1):50-62. doi:10.1017/s0963180115000286. |
| 49. Spence K. The best interest principle as a standard for decision making in the care of neonates. J Adv Nurs. 2000;31(6):1286-92. |
| 50. Taylor HJ. WHAT ARE 'BEST INTERESTS'? A CRITICAL EVALUATION OF 'BEST INTERESTS' DECISION-MAKING IN CLINICAL PRACTICE. Med Law Rev. 2016;24(2):176-205. doi:10.1093/medlaw/fww007. |
| 51. Tuckett AG. On paternalism, autonomy and best interests: telling the (competent) aged-care resident what they want to know. International journal of nursing practice. 2006;12(3):166-73. doi:10.1111/j.1440-172X.2006.00565.x. |
| 52. Walton R. The Best Interests of the Child. The British Journal of Social Work. 1976;6(3):307-13. |
| 53. Welie JV. Living wills and substituted judgments: a critical analysis. Med Health Care Philos. 2001;4(2):169-83. |
| 54. Wendler D. Are physicians obligated always to act in the patient's best interests? J Med Ethics. 2010;36(2):66-70. doi:10.1136/jme.2009.033001. |
| 55. Wilkinson D. Is It in the Best Interests of an Intellectually Disabled Infant to Die? Journal of Medical Ethics. 2006;32(8):454-9. |
| 56. Willmott L, White B, Smith MK, Wilkinson DJ. Withholding and withdrawing life-sustaining treatment in a patient's best interests: Australian judicial deliberations. The Medical journal of Australia. 2014;201(9):545-7. |
| 57. Wilson J. Patients' wants versus patients' interests. J Med Ethics. 1986;12(3):127-32. |

### Analysis

In order to capture the rich web of arguments emerging from the literature I began inductive coding of the sources using thematic analysis. The method involves first reading and familiarising oneself with the sources, then undertaking line by line coding using NVivo 12 software. Codes were inductively developed to group key ideas, concepts and arguments within multiple sources. As codes matured, they were grouped into broader themes, which were written up. Once themes were written and established, they were checked against the original sources to ensure they correctly represented them. 32 codes were developed from which three themes emerged.
